# Nucleic Acid-Induced Signaling in Chronic Viral Liver Disease

**DOI:** 10.3389/fimmu.2020.624034

**Published:** 2021-02-05

**Authors:** Armando Andres Roca Suarez, Barbara Testoni, Thomas F. Baumert, Joachim Lupberger

**Affiliations:** ^1^ INSERM, U1110, Institut de Recherche sur les Maladies Virales et Hépatiques, Strasbourg, France; ^2^ Université de Strasbourg, Strasbourg, France; ^3^ INSERM U1052, CNRS UMR-5286, Cancer Research Center of Lyon (CRCL), Lyon, France; ^4^ University of Lyon, Université Claude-Bernard (UCBL), Lyon, France; ^5^ Institut Hospitalo-Universitaire, Pôle Hépato-digestif, Nouvel Hôpital Civil, Strasbourg, France; ^6^ Institut Universitaire de France (IUF), Paris, France

**Keywords:** hepatitis B virus, hepatitis C virus, hepatocellular carcinoma, viral sensing, signaling, inflammation

## Abstract

A hallmark for the development and progression of chronic liver diseases is the persistent dysregulation of signaling pathways related to inflammatory responses, which eventually promotes the development of hepatic fibrosis, cirrhosis and hepatocellular carcinoma (HCC). The two major etiological agents associated with these complications in immunocompetent patients are hepatitis B virus (HBV) and hepatitis C virus (HCV), accounting for almost 1.4 million liver disease-associated deaths worldwide. Although both differ significantly from the point of their genomes and viral life cycles, they exert not only individual but also common strategies to divert innate antiviral defenses. Multiple virus-modulated pathways implicated in stress and inflammation illustrate how chronic viral hepatitis persistently tweaks host signaling processes with important consequences for liver pathogenesis. The following review aims to summarize the molecular events implicated in the sensing of viral nucleic acids, the mechanisms employed by HBV and HCV to counter these measures and how the dysregulation of these cellular pathways drives the development of chronic liver disease and the progression toward HCC.

## Introduction

The accelerating technological developments in biomedical and genomic research have made us aware of the enormous variety of viruses present in our environment and within ourselves (1, 2). Moreover, this has also given us a glimpse into our own past, as every biologic replication system with appreciable complexity co-evolves with parasites and at the same time develops mechanisms of resistance to them (3, 4). An important evolutionary milestone in vertebrates was the development of interferons (IFNs). IFN types I-III signal *via* the Janus kinase/signal transducer and activator of transcription (JAK/STAT) pathways, triggering a series of sophisticated antiviral defense mechanisms (5). These consist on the induction of a complex expression pattern comprising a myriad of interferon-stimulated genes (ISGs), which promote an inflammatory state aimed to counter the viral presence or tilt the cellular balance toward apoptosis if the infection is not resolved (6). As this response needs to be transient in order to avoid excessive tissue damage, IFN signaling pathways are tightly regulated by multiple feedback mechanisms, which include members of the suppressor of cytokine signaling (SOCS) family among others (7). IFNs are induced by certain types of exogenous nucleic acids (8). DNA and RNA are universal molecules in biology and thus any innate immune defense system based on the detection of viral nucleic acids must be capable of differentiating between the ones belonging to the host and ones that are foreign to it (9). Now, more than thirty years after this hypothesis was originally put forward, there has been substantial progress in the identification of these nucleic acid sensors and the understanding of their mechanisms of action (10).

Here, we explore these cellular receptors and their downstream signaling pathways in the context of the two major etiologies of chronic liver disease: hepatitis B (HBV) and hepatitis C virus (HCV). Patients with chronic viral hepatitis present a considerably increased risk of developing hepatic complications such as cirrhosis and hepatocellular carcinoma (HCC), with estimates suggesting that more than 1.4 million deaths each year are associated with these diseases (11, 12). HBV is a small noncytopathic DNA virus from the *Hepadnaviridae* family, and HCV is a single-stranded RNA virus from the *Flaviviridae* family (13, 14). Both exclusively hepatotropic viruses represent a tremendous global health burden with more than 320 million people chronically infected (15, 16). In this review, we introduce this topic with a general description of the cellular components implicated in the sensing of viral nucleic acids. This is followed by exploring the role of such sensors in the context of HBV and HCV infection and the viral strategies aimed to evade innate immune responses. Finally, we address the potential implications arising from the dysregulation of nucleic acid-sensing pathways as a driving component in the development and progression of HCC.

## Signaling Pathways Implicated in Viral Nucleic Acid Sensing

Pattern recognition receptors (PRR) implicated in the sensing of nucleic acids as pathogen-associated molecular patterns (PAMPs) can be divided in two main categories according to their mechanism of action (i.e., indirect or direct antiviral activity). The first category comprises PRRs belonging to the Toll-like receptor (TLR) and RIG-I–like receptor (RLR) families that are involved in RNA sensing, and a series of DNA sensors. These molecules induce the activation of transcription factors that favor the expression of cytokines such as type I IFN, ISGs and chemokines that recruit immune cells to the site of infection (17). In addition, these sensors can induce diverse types of programmed cell death such as apoptosis and pyroptosis in order to limit the spread of the infectious process (18). The second category of receptors includes nucleic acid sensors that possess direct antiviral activity, which is aimed against viral replication, translation or virion assembly (19, 20). Typically, the expression of such sensors is secondary to the production of IFNs or PRR signaling.

**Figure 1:**
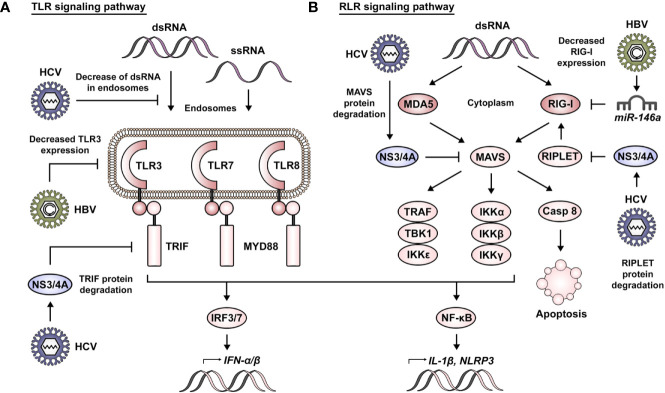
RNA sensing and viral manipulation of TLR and RLR signaling pathways. **(A)** Foreign RNA sensing is mediated by TLR3, 7, and 8 in the endosomal compartment. These signals are transduced *via* TRIF in the case of TLR3 and by intermediary of MYD88 for TLR7 and TLR8. Their activation leads to the production of type I IFNs *via* IRF3/7 and to the expression of other genes such as IL-1β and NLRP3 *via* the NF-κB pathway. HBV has been reported to impair TLR signaling pathways *via* decreased expression of TLR3. Similarly, HCV affects TLR signaling by decreasing the presence of dsRNA in endosomes and *via* NS3/4A-mediated degradation of TRIF. **(B)** Foreign RNA sensing is mediated by MDA5 and RIG-I in the cytoplasmic compartment. K63-linked ubiquitination of RIG-I by RIPLET is necessary for its activation. MDA5 and RIG-I signals converge in the activation of MAVS and are carried *via* TRAF, TBK1 and IKKϵ for the induction of IFNs, or *via* IKKα, IKKβ, and IKKγ for induction of the NF-κB pathway. Alternatively, MAVS signaling can also induce apoptosis *via* activation of caspase 8. HBV has been reported to impair RLR signaling pathways *via miR-146a*–mediated downregulation of RIG-I. Similarly, HCV affects RLR signaling *via* NS3/4A-mediated degradation of RIPLET and MAVS.

RNA sensors of the TLR family include TLR3, TLR7, and TLR8, with the three of them located at the endosomal membrane (21) (**Figure 1A**). In this context, TLR3 is activated by short double-stranded RNA (dsRNA) of 40 to 50 bp (22). TLR7 and TLR8 detect polyU and GU-rich stretches of nucleic acid, this being mainly dsRNA for TLR7 and exclusively single-stranded RNA (ssRNA) for TLR8 (23–25). These signals are carried downstream *via* TIR domain-containing adaptor-inducing IFN-β (TRIF) in the case of TLR3 (26, 27), while TLR7 and TLR8 do so by intermediary of myeloid differentiation primary response protein 88 (MYD88) (24). Their activation leads to the production of type I IFNs *via* interferon regulatory factor 3 (IRF3)/IRF7, and to the expression of other genes such as interleukin 1 beta (IL-1β) and NOD-, LRR-, and pyrin domain-containing 3 (NLRP3) *via* the nuclear factor-κB (NF-κB) pathway (28, 29).

The RLR family of RNA receptors comprises retinoic acid-inducible gene I (RIG-I) and melanoma differentiation-associated 5 (MDA5) as main sensors of foreign RNA in the cytoplasm (**Figure 1B**). RIG-I has been described to sense both long and short dsRNA, with the particular context defining if this is dependent or not of 5′ modifications (30–33). Additionally, RIG-I has been reported to recognize cytoplasmic DNA (34). MDA5 is strongly activated by very long dsRNA (30). A third member of the family, laboratory of genetics and physiology 2 (LGP2), functions primarily as a regulator of RIG-I and MDA5 activity (35).

RIG-I- and MDA5-induced signaling converges in the activation of mitochondrial antiviral signaling protein (MAVS), a key component acting as a hub for the induction of an antiviral state in the cell (36). Lysine 63 (K63)-linked ubiquitination of RIG-I by RING finger protein leading to RIG-I activation (RIPLET) is necessary for its interaction with MAVS at the mitochondrial membrane (37, 38). Subsequently, MAVS signaling leads to its association with tumor necrosis factor (TNF) receptor-associated factor (TRAF) proteins, TANK binding kinase 1 (TBK1) or IκB kinase ϵ (IKKϵ) in order to trigger activation of the IRF3/IRF7 pathway. Alternatively, this can occur *via* its association with the IKKα, IKKβ, and IKKγ complex for induction of the NF-κB pathway. The transduction of these signals results in the production of type I and III IFNs or the expression of proapoptotic genes such as p53-upregulated modulator of apoptosis (PUMA). Moreover, MAVS signaling can also induce apoptosis *via* activation of caspase 8 (39). As a side note, is worth mentioning that an important tool in the study and characterization of these RNA-sensing pathways has been the use of polyinosinic–polycytidylic acid (poly(I:C)). Structurally, poly(I:C) resembles dsRNA and is able to induce the activation of PRRs, such as TLR3, MDA5, and RIG-I (40, 41).

**Figure 2:**
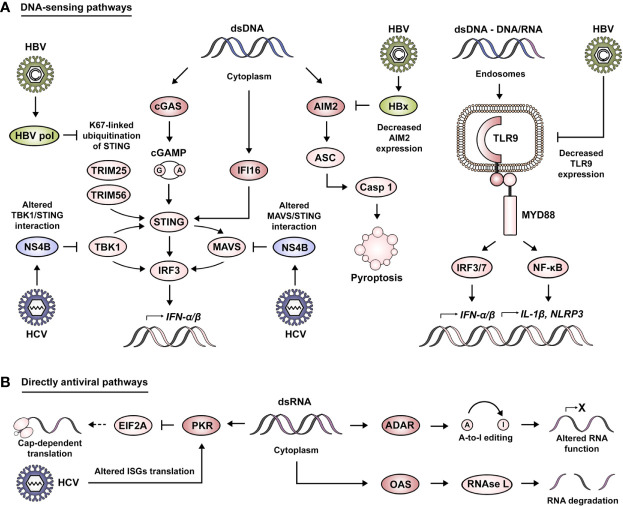
Viral manipulation of DNA-sensing and directly antiviral signaling pathways. **(A)** Foreign DNA sensing is mediated by cGAS, IFI16 and AIM2 in the cytoplasmic compartment. cGAS activation leads to the production of cGAMP, which acts as a second messenger to induce STING activation. cGAMP binding to STING induces its dimerization and K63 ubiquitination by TRIM25 and TRIM56. This leads STING to interact with TBK1 and transduce these signals *via* IRF3 or MAVS in order to produce type I IFNs. Moreover, STING can also be activated by IFI16. AIM2 sensing of cytosolic DNA results in its interaction with ASC, leading to the cleavage of caspase 1, the secretion of IL-1β and the induction of pyroptotic cell death. Foreign DNA sensing is mediated by TLR9 in the endosomal compartment. These signals are transduced by intermediary of MYD88 and IRF3/7 in order to produce type I IFNs, and to the expression of other genes such as IL-1β and NLRP3 *via* the NF-κB pathway. HBV has been described to impair DNA-sensing pathways *via* HBV pol-mediated alteration of K67-linked ubiquitination of STING, HBx-mediated degradation of AIM2 and the decrease of TLR9 expression. Similarly, HCV affects DNA sensing by altering TBK1/STING and MAVS/STING interaction *via* its viral protein NS4B. **(B)** Directly antiviral pathways are mediated by the action of PKR, ADAR, and OAS. PKR activation leads to the inhibition of mRNA translation *via* interference with EIF2A. HCV has been described to exploit PKR activity as a means to alter the translation of ISGs. OAS proteins lead to the activation of RNase L and the induction of RNA degradation. ADAR proteins mediate A-to-I editing, leading to the alteration of RNA structure, localization and coding capability.

Foreign DNA sensing is mediated by TLR9 in the endosomal compartment and by absent in melanoma 2 (AIM2), interferon gamma inducible protein 16 (IFI16) and cyclic GMP-AMP synthase (cGAS) in the cytosol (**Figure 2A**). TLR9 is responsible for the recognition of DNA containing unmethylated CpGs or short DNA/RNA hybrid molecules (42, 43). These signals progress through MYD88, IRF7 and NF-κB, as previously described for the other TLR family members. AIM2 seems to recognize dsDNA with a minimum length of 80 bp (44). cGAS senses dsDNA of approximately 20–40 bp, although these fragments can be shorter (≥12 bp) in the case of G-rich Y-form DNA (45, 46). IFI16 has been reported to recognize longer dsDNA molecules, with an optimal length of 150 bp (47). In the case of cGAS, its activation leads to the production of the dinucleotide cyclic GMP-AMP (cGAMP), which acts as a second messenger to induce downstream signaling *via* stimulator of interferon response cGAMP interactor (STING). The binding of cGAMP to STING induces its dimerization and K63 ubiquitination by the ubiquitin ligases tripartite motif protein 25 (TRIM25) and TRIM56 (48, 49). Activation of STING ultimately leads to its interaction with TBK1 and the transduction of these signals *via* IRF3 in order to produce type I IFNs (50, 51). Similarly, IFI16 binding to dsDNA leads to activation of the STING pathway (52). On the contrary, AIM2 sensing of cytosolic DNA results in its interaction with apoptosis-associated speck-like protein containing a CARD (ASC), a key adaptor protein in the formation of inflammasomes (53). This event leads to the cleavage of caspase 1, the secretion of IL-1β and the induction of pyroptotic cell death (54).

As mentioned before, the second category of nucleic acid receptors is characterized by having a direct antiviral action (**Figure 2B**). Some of the relevant examples of this class of molecules are protein kinase R (PKR), 2′-5′-oligoadenylate synthetase (OAS) and adenosine deaminase RNA specific (ADAR). PKR is activated by recognition of dsRNA with a length larger than 30 bp (55), leading to an inhibition of mRNA translation by interference with eukaryotic translation initiation factor 2 alpha (EIF2A) (56). OAS proteins recognize dsRNA with a similar length as PKR, but in this case, it leads to the synthesis of 2′-5′-oligoadenylate that acts as a second messenger for the activation of RNase L (latent) and the induction of RNA degradation (57). ADAR proteins bind to long dsRNA with complex secondary structures (58), a process that catalyzes the C6 deamination of adenosine to produce inosine (A-to-I editing) leading to the alteration of RNA structure, localization and coding capability (59).

## Sensing of HBV and HCV Nucleic Acids in Hepatocytes

Following acute infection, HCV induces a strong inflammatory response characterized by the expression of hundreds of ISGs (60). These transcriptional changes have been shown to correlate with HCV viral load, suggesting that the expression of ISGs is principally mediated by PAMPs that activate innate sensors such as the ones previously mentioned (61). In contrast, HBV has been classically described as a stealth virus due to its capacity not to induce an apparent immune response (62). This might be either due to the replication strategies that allow HBV to escape the innate immune response without being detected or due to direct suppressive action over IFN signaling cascades despite its detection by PRRs. In support of the former notion, it has been shown by the culture of *ex vivo* liver biopsies from HBV-infected patients, that presence of the virus does not hamper RNA-induced TLR and RLR signaling (63). Moreover, although DNA-sensing pathways such as cGAS/STING seem functionally activated, HBV infection suppressed cGAS expression and function in hepatocytes (64), but not in immune mediators such as Kupffer cells (65). However, as we will see in the following paragraphs, an increasing amount of evidence suggests that in spite of being a weak inducer of proinflammatory cytokines, HBV can be recognized by the innate immune system (66).

## TLR Signaling Pathways

Members of the TLR family, namely TLR3, 7 and 9 have been shown to play key roles mediating immune responses against HBV infection in an IFN-dependent manner (67). Similarly, HCV infection has been reported to induce the expression of TNF-α *via* TLR7/8 signaling. Consequently, TNF-α activates its receptor (i.e., TNFR1) and leads to the suppression of HCV *via* the expression of ISGs (68). The clinical relevance of TLR signaling in the context of these two hepatotropic viruses has been highlighted by the identification of single nucleotide polymorphisms (SNPs) present in the *TLR3* and *TLR9* genes, as they are associated with the clinical course of infection. Indeed, *TLR3* SNPs have been linked to a reduced likelihood of spontaneous HBsAg and HBeAg seroclearance and an increased risk of developing chronic HBV infection (69, 70). These SNPs are also relevant during HCV infection as they are associated with an increased risk of chronic infection and HCV-associated liver disease (70). In particular, *TLR9* SNPs have been associated with spontaneous HCV clearance in women, as the expression of TLR9 in this context was linked to the level of circulating estrogens (71).

## RLR Signaling Pathways

RIG-I activation during HBV infection results in a weak production of IFN-α/β in contrast to a marked induction of IFN-λ (72). Moreover, the substrate recognized by RIG-I was shown to be a 5′ stem loop in the HBV pregenomic RNA (pgRNA), a region described to contain the encapsidation (ϵ) sequence. This observation suggests a potential direct antiviral role of RIG-I *via* interference with binding of the HBV polymerase to the viral pgRNA (72). In contrast, HCV-induced RIG-I activation has been shown to produce a strong hepatic IFN-β expression, which is followed by the induction of an antiviral response mediated by ISGs (e.g., ISG54 and ISG56) (73). In this context, it has been observed that cytosolic HCV RNA is sensed by RIG-I, specifically at the HCV 3′ poly-U/UC sequence, the 5′ triphosphate of the uncapped HCV RNA and several short dsRNA regions (73, 74).

MDA5 also mediates the sensing of nucleic acids associated with the HBV infectious process (75). Indeed, HBV replication significantly increases the expression of MDA5 *in vivo*. Moreover, MDA5 overexpression induces a decrease of HBV RNA and encapsidated DNA *in vitro*. In mechanistic studies, the authors demonstrated that overexpression of MDA5 during HBV infection leads to IRF3 activation, NF-κB translocation to the nucleus and the subsequent expression of ISGs (e.g., MxA, OAS1, and CXCL10) (75). There is also evidence for a link between HCV and MDA5 (76). RIG-I and MDA5 activation by HCV occurs in a sequential and MAVS-dependent manner, as the IFN response is mediated by RIG-I at early stages of infection while the action of MDA5 takes place subsequently (77). Just like in the case of TLR components, the clinical relevance of MDA5 in HCV-associated disease has been shown by the observation that SNPs in the *IFIH1* gene (encoding for MDA5) are highly correlated with the resolution of HCV infection. Indeed, expression of these gene variants *in vitro* led to increased secretion of CXCL10 and IFN-λ3, concomitantly with a surge in the expression of other ISGs (e.g., IFN-β, ISG15 and ISG56) (78). Recent evidence suggests that LGP2 plays a role in strengthening MDA5-mediated innate immune responses against HCV infection. Indeed, LGP2 was shown to increase HCV RNA levels in association with MDA5 *via* its ATPase activity, leading to the expression of IFN-β and ISGs (79). On the contrary, other studies have reported that LGP2 negatively regulates these signaling pathways by interacting with TRAF family proteins and interfering with their ubiquitin ligase activity (80).

## DNA-Sensing Pathways

The role of cytoplasmic DNA sensors implicated in the induction of antiviral responses has been mostly described for HBV, with very few examples related to HCV infection. In this regard, the impact of two receptors in this category, IFI16 and AIM2, has been studied using woodchuck hepatitis virus (WHV) as a model system since it strongly resembles human HBV infection. In this model, expression levels of IFI16 and AIM2 were reported to be increased following acute infection with WHV. This tendency was consistent in the case of AIM2 following analysis of liver samples from chronically infected animals, but slightly decreased levels were observed for IFI16 (81). Interestingly, a subsequent report described that IFI16 is able to bind the HBV covalently closed circular DNA (cccDNA), particularly after IFN-α stimulation (82). Indeed, IFI16 overexpression was able to hamper the HBV cycle as shown by the decrease in hepatitis B surface antigen (HBsAg), hepatitis B e antigen (HBeAg), precore (preC)-pgRNA and HBV DNA. The expression level of IFI16 in human liver biopsies was significantly downregulated in patients with chronic HBV infection, negatively correlating between IFI16 and HBV preC-pgRNA. Moreover, taking into account that IFI16 seemed not to affect cccDNA quantity, this observation further suggested its repression at the transcriptional level. Indeed, the authors were able to demonstrate that IFI16 overexpression significantly decreased the levels of active histone marks and promoted the deposition of repressive ones in the HBV cccDNA minichromosome. Furthermore, recruitment of the acetyltransferase CREB-binding protein (CBP) to the minichromosome was impaired. Similarly, cccDNA in association with the deacetylases sirtuin 1 (SIRT1) and histone deacetylase 1 (HDAC1), as well as the lysine methyltransferase enhancer of zeste 2 polycomb repressive complex 2 subunit (EZH2) was significantly increased. The authors went even further and showed that the interferon stimulating responsive element (ISRE) sequence present in the HBV cccDNA was needed to interact with IFI16 and induce this epigenetic regulation (82).

Although, HBV DNA is protected by the capsid and thus not accessible to DNA sensors in the cytoplasm, cGAS can recognize extracted HBV DNA as foreign (83) and mount an immunostimulatory response *via* the cGAS/STING pathway in hepatic cells (84). A functional role of this pathway beyond sensing was suggested by Eloi Verrier and co-workers, demonstrating that transfection of HBV relaxed circular DNA (rcDNA) induces the expression of ISGs, and importantly, knockout or overexpression of cGAS resulted in a marked increase in HBV infection and impaired HBV cccDNA levels (64). In support of this, the use of a STING agonist in HBV-infected mice decreased viral load and the susceptibility to infection (85). Thus, although STING expression is low in hepatocytes (65), a functional link with HBV seems evident. For HCV, although not a DNA virus, cGAMP stimulation or STING overexpression has an inhibitory effect on viral replication (86), as discussed below in more detail.

## Directly Antiviral Pathways

In addition to its suppressing role on mRNA translation (87), PKR recognizes the HCV internal ribosome entry site (IRES) and contributes to an antiviral response at early stages of infection by interacting with MAVS and triggering ISG expression (88, 89). Moreover, based on the observation that the activity of PKR is negatively regulated by cyclophilin A (CypA) (90), it was recently reported that PKR signaling determines the potency of cyclophilin inhibitors (CypI) against HCV *via* engagement of IRF1 (91). Other directly antiviral pathways comprise OAS and ADAR1 proteins, which exhibit an intrinsic antiviral activity targeting HCV infection (92, 93). The p100 subunit of OAS3 presents an antiviral role against HCV in an RNase L-dependent manner (92). ADAR1 specifically targets HCV replication *via* A-to-I editing (94). ADAR1 also targets HBV infection. Guangyan Liu and co-workers have recently shown that *in vitro* overexpression of ADAR1 induces a decrease of HBV RNA and nucleocapsid-associated DNA. The mechanism suggested by the authors involves an ADAR1-mediated upregulation of *miR-122* leading to decreased levels of cyclin G1, a subsequent increase of p53 and ultimately reduced HBV RNA levels (95).

## Sensing of HBV and HCV Nucleic Acids in Hepatic Stromal Cells

Although our view of the liver has been traditionally hepatocyte-centric, recent developments in single-cell omics have opened new insights into the diversity of stromal components and cellular states present in the liver microenvironment (96, 97). This is highly relevant in context of viral liver diseases, as it has been shown for example that HBV and HCV particles or viral components can be detected in association with stromal cells, such as fibroblasts (98), lymphocytes (99), endothelial (100) and dendritic cells (101). Although HBV and HCV may not be able to replicate in these cell types (102, 103), their exposure to viral nucleic acids does induce the activation of pathways implicated in antiviral immune responses (104). In this regard, it has been reported that TLR-mediated antiviral activity is not limited to infected hepatocytes, but is also induced in hepatic stellate cells (HSCs) and peripheral blood mononuclear cells (PBMCs). Indeed, treatment of HepG2 cells with supernatants from poly(I:C)-stimulated Lx-2 cells (HSC-derived cell line) inhibits the release of HBeAg and HBsAg in an IFN-β-dependent manner (105). Likewise, direct contact between HCV-infected hepatocytes and type 2 myeloid dendritic cells (mDC2) leads to the detection of dsRNA by TLR3 and the production of IFN-λ (e.g., IL-28 and IL-29) (106). Moreover, it has been observed that stimulation of PBMCs with HCV RNA leads to the production of TNF-α *via* TLR7/8 and that supernatants from monocytes or plasmacytoid dendritic cells (pDCs) stimulated with HCV RNA are able to reduce viral replication *in vitro* (107). These results are in line with a previous report by Marlène Dreux and co-workers showing how HCV infection in hepatocytes induces the release of exosomes carrying HCV RNA, which is recognized by TLR7 in pDCs (108). In addition to TLR-mediated signaling, the cGAS/STING pathway is also activated in myeloid cells following their stimulation with HBV DNA *in vitro* (84). Although its *in vivo* relevance remains to be established, cGAS/STING signaling in non-parenchymal cells is of potential clinical importance as dysregulation of this pathway is associated with liver pathogenesis (109, 110) (as discussed later).

## HBV and HCV Strategies to Evade Nucleic Acid-Induced Antiviral Responses

The innate immune system is a result of our co-evolution with pathogens. In response to the evolutionary pressure from the immune system, viruses have developed a wide variety of elaborated evasion mechanisms in order to prevent their elimination by the host (111). As HBV and HCV induce chronic liver infection, both viruses are no exception to this rule.

Immune evasion strategies relay on the ability of the virus to passively go unnoticed or/and actively attenuate downstream signaling events leading to a blunted IFN response. Manipulation of TLR3 signaling by HBV and HCV represents a clear example of both (**Figure 1A**). In this context, a recent report has shown that HCV dsRNA is released in extracellular vesicles, leading to a decreased activation of TLR3 in HCV-infected cells and allowing viral escape from the innate immune system (112). Of interest, HCV also actively interferes with downstream components of this pathway, e.g., by reducing the abundance of TRIF *via* NS3/4A-mediated proteolysis. This results in a delayed expression of ISGs (e.g., ISG15 and ISG56) and the establishment of a persistent infection (113). Although HBV is remarkably proficient in escaping immune detection *via* passive mechanisms, this virus has also been shown to actively drive the suppression of defense mechanisms. Indeed, the observation of a biphasic induction of ISGs following HBV infection suggests a feedback suppression (103). In this regard, HBV impairs TLR signaling by suppressing TLR3 expression in chronically infected patients (114).

The interference of HBV and HCV with RIG-I function is elaborated and complex (**Figure 1B**). Hou *et al*., revealed that HBV infection induced the downregulation of RIG-I and IFN-β in HepG2 cells and human HBV tissues. Furthermore, the authors uncovered an HBV-induced upregulation of *miR-146a* mediating RIG-I suppression and that inhibition of *miR-146a* accelerated HBV clearance *in vivo* (115). This is consistent with previous observations reporting that HBV can abort cell intrinsic immunity in hepatocytes, with weak, transient IFN-α/β and IL-6 induction. In addition to the HBV-induced repression of RIG-I, this is a consequence of impaired MDA5 and TLR3 pathways during infection (116). Interestingly, HCV inactivates RIG-I signaling *via* the proteolytic activity of its viral protein NS3/4A. NS3/4A inhibits the induction of IFN-β *via* its localization to the mitochondrial membrane, where it is able to cleave MAVS and thus favor HCV evasion from the immune response (117). This is supported by translational studies demonstrating the presence of cleaved MAVS in liver biopsies from HCV- but not HBV-infected patients (118). Indeed, MAVS cleavage is particularly marked in HCV NS3-expressing hepatocytes (119), along with the cytoplasmic, and therefore inactive, form of IRF3 (120). Moreover, the NS3/4A protease complex antagonizes the cellular E3 ubiquitin ligases, TRIM25 and/or RIPLET, thereby also inhibiting RIG-I ubiquitination and thus its activation (121). Finally, the sequential activation of RIG-I and MDA5 can be inhibited by the action of HCV NS5A, as demonstrated both *in vitro* and *in vivo* (77).

Even though the main role attributed to the STING pathway is the sensing of DNA, it has also been implicated during HCV infection, which produces no DNA intermediate (**Figure 2A**). In this regard, HCV protein NS4B impairs IFN-β induction by altering the interaction between STING and TBK1 as a means of immune evasion (122). Moreover, NS4B competes with MAVS for binding to STING on mitochondria-associated membranes. This interaction results in the displacement of MAVS from the RIG-I/MAVS/STING complex and prevents the downstream transduction of signals *via* IRF3 (123). In agreement with these findings, Guanghui Yi and co-workers demonstrated that the NS4B-mediated STING suppression was more pronounced when using NS4B from HCV genotype 2a (86). For HBV infection, it has been reported that the HBV polymerase inhibits cGAS/STING signaling and the subsequent activation of IRF3 by disrupting K63 ubiquitination of STING, thus preventing the production of IFN-β (124). HBV infection also impairs AIM2 expression *via* HBx. It prolongs the half-life of EZH2, thus favoring its recruitment to the AIM2 promoter, and subsequently blocking its expression (125). Regarding endosomal DNA sensing, it has been demonstrated how the presence of HBV virions and subviral particles specifically downregulates TLR9 at the mRNA and protein level, leading to significantly reduced levels of CpG-induced IFN-α in both pDCs and PBMCs. This is further supported by the observation that TLR9 levels are drastically reduced in chronic HBV patients (126, 127).

As previously mentioned, HCV-induced phosphorylation of PKR leads to the inactivation of EIF2A and ultimately to a halt in protein production as a means of antiviral response (87) (**Figure 2B**). Impaired translation of mRNAs is exploited by HCV in order to overcome the antiviral defense in hepatocytes, mainly by inhibiting the translation of ISGs (128).

## Dysregulation of Nucleic Acid-Sensing Components Implicated in HCC Development

**Figure 3:**
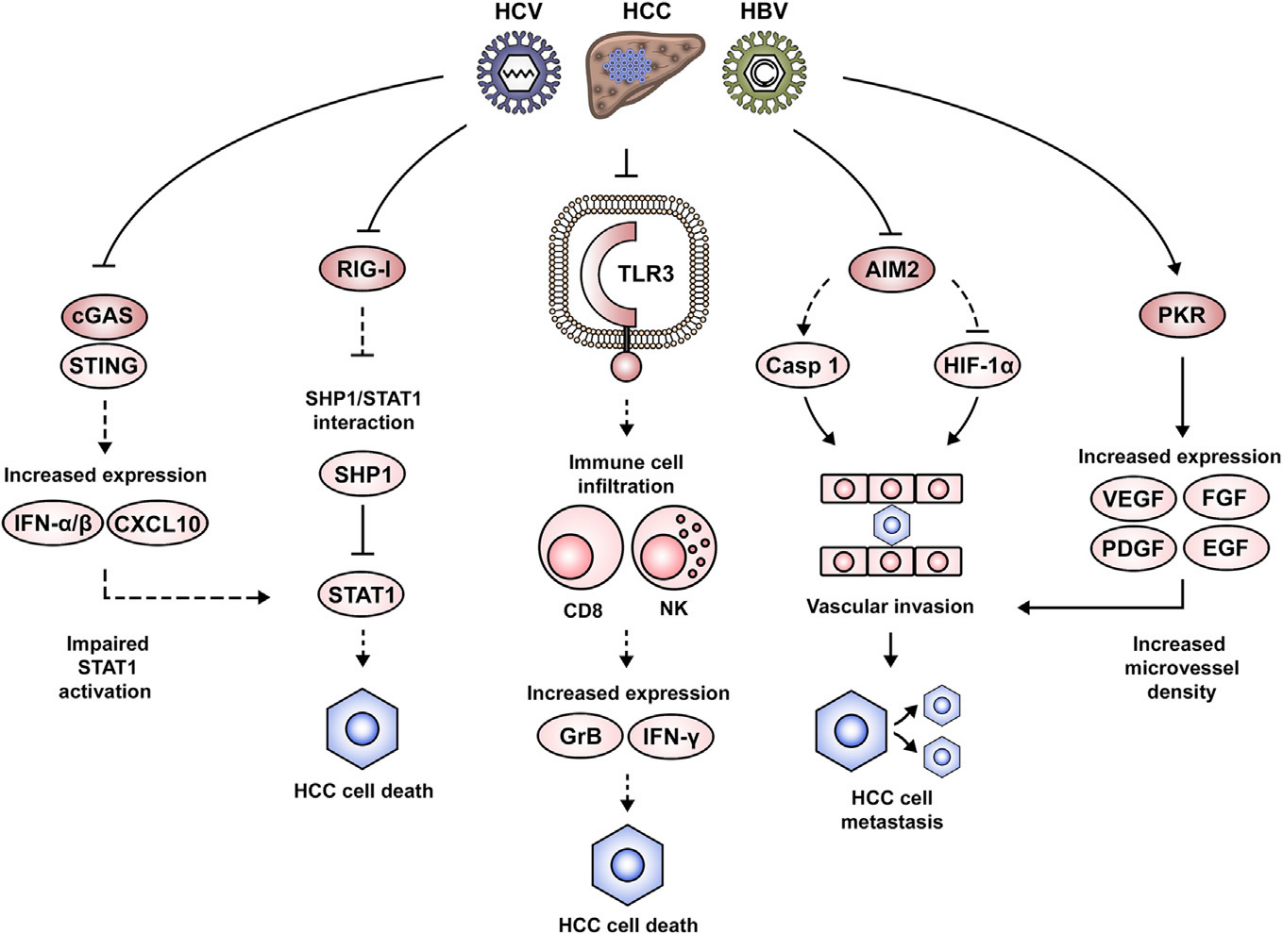
Dysregulation of nucleic acid-induced signaling during HCC development. Altered nucleic acid-sensing pathways associated with HCC pathogenesis include cGAS/STING, RIG-I, TLR3, AIM2 and PKR. Impairment of RIG-I expression favors STAT1 interaction with its negative regulator SHP1, thus altering STAT1-mediated proapoptotic signaling. Similarly, the suppression of IFN signaling *via* the cGAS/STING pathway leads to an altered STAT1 activation. Downregulation of TLR3 in the context of HCC leads to an impaired attraction/activation of immune cells to the tumor microenvironment, the failure to induce a gene expression pattern that mediates cytotoxicity (e.g., GrB, IFN-γ) and the subsequent clearance of cancer cells. AIM2 downregulation alters normal formation of the AIM2 inflammasome and favors expression of HIF-1α, leading to the development of malignant cellular characteristics such as vascular invasion and lymph node metastasis. Activation of PKR in tumor lesions favors the expression of growth factors such as VEGF, PDGF, FGF, and EGF, which favor angiogenesis and potentially the development of vascular invasion and metastasis.

An important observation with relevance to virus-associated pathogenesis is that a high number of nucleic acid PAMP receptors are also able to sense host nucleic acids as damage-associated molecular patterns (DAMPs) (129). DAMPs are central players in tissue repair, as they alert the organism about injury, stimulate an inflammatory response and promote the activation of organ regenerative pathways (130). For example, MAVS and STING deficiency results in impaired hepatocyte proliferation and a delayed recovery of liver mass following partial hepatectomy (131). Therefore, it is not surprising that due to continuous viral stimulation or suppression in a chronic inflammatory microenvironment, the dysregulation of innate immune components contributes to liver disease progression and complications including HCC (**Figure 3**).

TLRs activate inflammatory signaling pathways in the liver, which if persistent, can drastically affect the delicate balance between cell death and survival (132). In this context, the proinflammatory and proapoptotic role of TLR3 is evident in poly(I:C)-stimulated cells, which can be rescued by TLR3 silencing (133). Additional details regarding the mechanism behind this observation came from the finding that TLR3 was colocalized with granzyme B (GrB), suggesting an expression predominantly in natural killer (NK) cells. Indeed, analysis of liver samples from an HCC patient cohort (68% of viral etiology) showed that TLR3 expression was correlated with the hepatic infiltration of NK and CD8 T cells. Moreover, *in vitro* stimulation of TLR3 promoted NK cell activation, expression of IFN-γ and GrB and cytotoxicity against HCC cells (133). These observations are in line with the analysis of HCC patient samples, which showed the infiltration of CD8 T cells being positively correlated with cell apoptosis and negatively correlated with cell proliferation. Therefore, the authors suggest that the tumor suppressing effects of TLR3 may come from the induction of hepatocyte death and the attraction/activation of immune cells to the tumor microenvironment. These results could be one of the mechanisms explaining the association between low TLR3 expression and a lower patient survival from HCC (133). Similar results were obtained by Marc Bonnin and co-workers, demonstrating that TLR3 expression was downregulated in HCC lesions as compared to adjacent tissues, and that this was associated with a poor prognosis. Moreover, the study of a TLR3-deficient mouse model allowed to conclude that hepatocarcinogenesis was indeed accelerated in the absence of TLR3. However, an association with immune cell infiltration was not evident in this study (134). The role of TLR7 in HCC is debated. While TLR7 expression is downregulated in tumor lesions of HBV- and HCV-associated HCC as compared to non-viral etiologies (135), another study finds TLR7 overexpressed in HCC lesions as compared to tissues from cirrhosis or viral hepatitis. Moreover, inhibition of TLR7 with a TLR7/9 inhibitor (IRS-954) hampered cell proliferation (136). In agreement with these findings, a positive TLR9 immunostaining had previously been associated with a poor HCC prognosis, which may suggest that TRL7/9 signaling is promoting cancer cell proliferation and survival (137).

The RLRs RIG-I and MDA5 have also been implicated in the pathogenesis of HCC. This has been demonstrated in paired liver biopsies from an HCC cohort (90% HBV-associated) (138). The study concluded that RIG-I was significantly downregulated in HCC lesions as compared to the adjacent tissues. The suggested mechanism involves an epigenetic dysregulation of the *RIG-I* gene, as it presented reduced levels of H3K4me3, but increased H3K9me3 and H3K27me3 marks (138). Taking into account that RIG-I maintains IFN-induced proapoptotic signaling *via* STAT1, the authors revealed that RIG-I prevents STAT1 engagement with its negative regulator Src homology region 2 domain-containing phosphatase-1 (SHP1). Thus, impaired RIG-I function indirectly promotes cell survival. Phenotypically, RIG-I deficiency is associated with the development of HCC, as it favored the incidence, number and size of HCC tumors in a mouse model. Moreover, impaired RIG-I expression was associated with a lower patient survival from HCC (138). These results are in agreement with previous observations, showing that stimulation of RIG-I and MDA5 expression with poly(I:C) in HepG2 cells limited their proliferative capacity *in vivo* (35). Downstream of RLR family members, expression of NLRP3 inflammasome components are impaired in HCC tumor tissues, i.e., ASC, caspase 1 and IL-1β, which inversely correlated with tumor grade and clinical stage (139).

DNA-sensing components linked to HCC development include IFI16, AIM2 and the cGAS/STING pathway. IFI16 protein expression is significantly decreased in HCC tissues (140). *In vitro* experiments suggest that IFI16 may exhibit a tumor-suppressing role, as its overexpression inhibited colony formation and induced cell apoptosis. Mechanistic studies suggested the capacity of IFI16 to induce p53 expression and its activation by promoting serine 15 (S15) phosphorylation (140). *AIM2* transcripts are specifically reduced in tumor tissue of paired liver biopsies from HBV-associated HCC. This impaired *AIM2* expression was associated with higher alpha-fetoprotein (AFP) levels, vascular invasion, poor tumor differentiation and lymph node metastasis. Moreover, patients with low *AIM2* mRNA levels showed shorter overall and disease-free survival times (125). These results are in agreement with the previous findings of Xiaomin Ma and co-workers, demonstrating AIM2 downregulation in HCC tissues as compared to the non-tumoral areas (141). Loss of AIM2 expression correlated with advanced tumor stages and metastasis. *In vitro* experiments demonstrated that AIM2 overexpression hampered cell proliferation, colony formation and invasion, while AIM2 silencing promoted an opposite more aggressive phenotype. The cellular signaling alterations arising from AIM2 dysregulation were also explored, demonstrating that increased AIM2 expression led to a significantly increased caspase 1 activation and IL-1β cleavage, indicating that the AIM2 inflammasome was formed and active. Moreover, blocking the inflammasome formation reversed the malignant characteristics. Additionally, AIM2 silencing enhanced hypoxia-inducible factor 1 alpha (HIF-1α) activity, which raised the possibility of the AIM2/HIF-1α axis contributing to the previously observed malignant cell characteristics (141). An interesting observation related to the involvement of DNA-sensing pathways in HCC development was made by Martin K. Thomsen and colleagues (109). The authors reported that increased STING expression rarely occurs in transformed hepatocytes and the response to STING agonists takes place primarily in non-parenchymal liver cells (e.g., Kupffer cells). Indeed, stimulation of a macrophage-like cell line (i.e., THP-1) with the STING ligand cyclic adenine monophosphate- inosine monophosphate (cAIMP) led to an enhanced expression of T cell-attracting chemokines (e.g., CXCL10), IFN-α/β and NF-κB activation. This molecular expression pattern following STING activation was identified as a driver for the induction of apoptosis, autophagy and the overall immune response. The pathological consequences of impaired hepatic STING function were explored by the authors using a liver cancer mouse model, which showed that STING-deficient animals presented larger tumors as compared to the wild-type mice. Tumor characterization revealed decreased phospho-STAT1 and phospho-STAT3 levels, suggesting an altered immune response. Additionally, it was demonstrated that treatment with cAIMP led to the development of smaller tumor nodules due to an enhanced apoptotic cell death, as suggested by increased levels of cleaved caspase 3-positive cells (109). The previously discussed report is in agreement with the results obtained by Dou *et al*., showing that STING activity favored hepatic inflammation and a DNA damage-induced senescence-associated secretory phenotype (SASP). Indeed, STING-deficient mice exhibited an impaired expression of SASP genes. Moreover, STING-deficient mice presented NRas-positive intrahepatic tumors, which was not the case in the wild-type animals. These results were confirmed by rescuing STING in the liver of these mice, resulting in the restoration of cytokine levels and immune-mediated clearance (110).

Among the nucleic acid sensors with direct antiviral action, PKR and ADAR have been implicated in HCC pathogenesis (142–144). A PKR inhibitor (C16) reduced cell division in Huh7 cells and markedly decreased liver tumor growth in a mouse model. Moreover, it occurred that microvessel density of such tumors was decreased during PKR inhibition. Indeed, *in vitro* experiments revealed that angiogenesis-relevant growth factor expression—i.e., vascular endothelial growth factor A (VEGF-A), VEGF-B, platelet-derived growth factor A (PDGF-A), PDGF-B, fibroblast growth factor 2 (FGF-2), epidermal growth factor (EGF), and hepatocyte growth factor (HGF)—was significantly downregulated by PKR inhibition (142). These observations are in line with the results reported following analysis of a human HCC cohort (100% HCV-associated), showing increased activity of PKR in tumor samples as compared to cirrhotic tissues (143). ADAR proteins (ADAR1 and ADAR2) are linked to HCC but seem to exhibit different roles during hepatocarcinogenesis. Immunohistochemistry and quantitative PCR (qPCR) in an HCC patient cohort identified upregulated ADAR1 but downregulated ADAR2 levels in HCC lesions as compared to the corresponding adjacent tissues. This ADAR expression pattern associated with increased incidence of liver cirrhosis and tumor recurrence with shorter disease-free survival times. These results were validated *in vitro* and *in vivo*, showing that overexpression of ADAR1 and ADAR2 was able to accelerate or inhibit tumor growth, respectively (144).

Taken together, the available data suggest that nucleic acid-sensing pathways are not only relevant as antiviral mechanisms against HBV and HCV infection, but—if chronically dysregulated—also contribute to the development and progression of virus-associated hepatic complications.

## Perspectives

As we have explored in this review, nucleic acid-induced inflammation represents a complex signaling pattern designed to push the cell into a transient state of emergency following the detection of viral pathogens. Viruses causing chronic hepatitis like HBV and HCV have developed elaborated strategies to attenuate and divert these antiviral responses contributing to failure of viral clearance and a chronic inflammatory state. Consequently, the chronic dysregulation of the inflammatory response is an important factor in liver disease progression toward HCC. It also emerges that an integrated view on not only infected hepatocytes but also on their complex interplay with stromal cell components will be central to understand viral pathogenesis (145). The microenvironment of chronically-infected livers in single-cell resolution is still poorly characterized. This type of analysis has already been performed in context of nonalcoholic steatohepatitis (NASH), leading to the identification of a cell population tightly linked to disease severity, termed NASH-associated macrophages (NAMs) (146). Therefore, the integrated analysis of multiomics single-cell data could provide a unique opportunity to grasp the impact of nucleic acid inflammatory pathways in each hepatic cell population following viral infection (147). Moreover, such data would be highly relevant for drug discovery and the development of biomarkers for stratification of patients that may benefit from targeted interventions (148). However, access to fresh liver tissues from patients with HCV or HBV viremia, which are required for this kind of analysis, gets more and more challenging as an increasing number of patients currently receive antiviral treatment.

Despite efficient antiviral regiments allowing to control (HBV) and cure (HCV) chronic viral hepatitis in 2020, the risk of HCC cannot be fully eradicated, especially in patients with advanced liver disease. For HCV, accumulating evidence highlights a viral footprint in the host genome maintaining a persistent proinflammatory and prooncogenic environment even after viral cure (149, 150). Thus, the understanding of virus-specific and common evasion mechanisms from the antiviral inflammatory response, genomic imprinting, and the characterization of pro-oncogenic signaling persisting after HCV cure is key to identify targets for future chemopreventive and antifibrotic strategies to help patients at elevated liver cancer risk.

While for HCV, the future challenges are mostly emphasized in the chemoprevention, for HBV, novel antiviral concepts are urgently needed to cure viral infection, potentially, embodied by host-targeting agents (HTAs) aimed to boost innate immunity. This approach is supported by several lines of evidence, including the fact that patients who achieve control of HBV infection do so *via* an efficient immune response. Moreover, the small size of the HBV genome imposes a limit to the possible drugs that can be designed against it and its encoded viral proteins (151). Therefore, HTAs have appeared as a viable option to overcome this and similar issues. In this context, the use of Riboxxol (TLR3 agonist) has been reported to decrease intracellular HBV DNA, as well as extracellular HBeAg and HBsAg *in vitro* (152). Similarly, Selgantolimod (TLR8 agonist) has shown encouraging results in the WHV model, as it was well tolerated, reduced intrahepatic WHV RNA and DNA levels and favored the development of anti-WHsAg antibodies (153). Selgantolimod is currently being evaluated in phase II trials, in order to assess its safety, tolerability and antiviral activity during chronic HBV infection (NCT03615066 and NCT03491553).

The aforementioned examples represent only a fraction of the potential that this approach has, as future compounds targeting additional nucleic acid sensors may as well be clinically relevant. In this regard, a close collaboration between academic research and industry will be necessary to accelerate drug discovery and evaluate their application as single or combination therapies (154). This will provide us with the possibility to expand our current therapeutic options and ameliorate the unmet medical need that chronic liver disease represents.

## Author Contributions

All authors contributed to the article and approved the submitted version.

## Funding

This work was supported by the European Union (EU H2020 HEPCAR 667273 to JL and TFB), the French Cancer Agency (TheraHCC2.0 IHU201901299 to JL and TFB), the Agence Nationale de Recherche sur le Sida et les hépatites virales (ANRS ECTZ103701 and ECTZ131760 to JL, ANRS ECTZ75178 to BT), the French Fondation pour la Recherche Médicale (FDT201805005763 to AARS), the Fondation de l’Université de Strasbourg (HEPKIN) (TBA-DON-0002), and the Inserm Plan Cancer 2019-2023. This work has benefitted from support by the Initiative of excellence IDEX-Unistra (ANR-10-IDEX-0002-02) and has been published under the framework of the LABEX ANR-10-LAB-28 (HEPSYS). Inserm Plan Cancer, IDEX, and LABEX are initiatives from the French program “Investments for the future”.

## Conflict of Interest

The authors declare that the research was conducted in the absence of any commercial or financial relationships that could be construed as a potential conflict of interest.
